# Identifying potential provenances for climate-change adaptation using spatially variable coefficient models

**DOI:** 10.1186/s12862-024-02260-z

**Published:** 2024-05-28

**Authors:** Marieke Wesselkamp, David R. Roberts, Carsten F. Dormann

**Affiliations:** 1 Department of Biometry and Environmental System Analysis, University of Freiburg, Tennenbacher Straße 4, Freiburg, 79106 Germany; 2 InnoTech Alberta, 3608 – 33 Street NW, Calgary, AB T2L 2A6 Canada; 3 Alberta Biodiversity Monitoring Institute, 1-107 Centennial Centre for Interdisciplinary Studies (CCIS), University of Alberta, Edmonton, AB T6G 2E9 Canada

**Keywords:** Climate-change adaptation, Douglas-fir, Ecotype, Genetic variation, Identifying ecotypes, Provenance tests, *Pseudotsuga menziesii*, Spatially variable coefficient models

## Abstract

**Background:**

Selection of climate-change adapted ecotypes of commercially valuable species to date relies on DNA-assisted screening followed by growth trials. For trees, such trials can take decades, hence any approach that supports focussing on a likely set of candidates may save time and money. We use a non-stationary statistical analysis with spatially varying coefficients to identify ecotypes that indicate first regions of similarly adapted varieties of Douglas-fir (*Pseudotsuga menziesii* (Mirbel) Franco) in North America. For over 70,000 plot-level presence-absences, spatial differences in the survival response to climatic conditions are identified.

**Results:**

The spatially-variable coefficient model fits the data substantially better than a stationary, i.e. constant-effect analysis (as measured by AIC to account for differences in model complexity). Also, clustering the model terms identifies several potential ecotypes that could not be derived from clustering climatic conditions itself. Comparing these six identified ecotypes to known genetically diverging regions shows some congruence, as well as some mismatches. However, comparing ecotypes among each other, we find clear differences in their climate niches.

**Conclusion:**

While our approach is data-demanding and computationally expensive, with the increasing availability of data on species distributions this may be a useful first screening step during the search for climate-change adapted varieties. With our unsupervised learning approach being explorative, finely resolved genotypic data would be helpful to improve its quantitative validation.

**Supplementary Information:**

The online version contains supplementary material available at 10.1186/s12862-024-02260-z.

## Introduction

Local adaptation strategies to current environmental changes—be it climatic, with respect to atmospheric pollution with NO_x_, CO_2_ and SO_x_, or to pests and diseases—heavily rely on identifying species, breeds and lineages that are best suited for such conditions and hence maintain higher yields in forests, croplands and animal production [1–4]. Widespread species such as common forest trees live under differing climatic conditions throughout their range. These conditions exert selective pressure, often leading to specific local adaptation to habitat conditions [2, 5]. In trees, this may result in substantial variation in cold hardiness or drought resistance among populations of the same species [1, 6–9]. Adaptive differences may eventually become genetically encoded and analysis of such intra-specific variation thus can reveal geographic patterns that are related to external selective factors [1, 10].

Populations of a species descending from different provenances can be tested for climatic adaptation by their growth performance under new conditions [11–13]. However, such growth trials are extremely time consuming, and tree provenance trials initiated in the 20th century are now still analysing young trees [14–16]. Genetic investigations can aid the identification of climatically adapted populations by delimiting ecotypes, which are then subjected to experimental physiological investigations (e.g. [17]). Yet, there may be hundreds of seed sources to choose from and being able to pre-select regions from which to sample specimen for genetic and/or physiological investigations could save both time and money.

Douglas-fir (*Pseudotsuga menziesii*) is a wide-ranging tree species, known to be closely adapted to its environment [18]. Due to the high economic and ecological importance, its characteristics have been well analysed in many studies and data on its distribution and characteristics are available. Its habitat extends from the coast of British Columbia (Canada), through the Rocky Mountains and adjacent mountainous regions down to the Mexican mountains. Two varieties are recognized: *Pseudotsuga menziesii* var. *menziesii*, growing along the Pacific coast line from British Columbia into California and *Pseudotsuga menziesii* var. *glauca*, inhabiting the interior mountain ranges. Since it is highly adaptive and productive, Douglas-fir has also been introduced as a non-native species to European forests [19]. Long-term provenance trials have been carried out in order to monitor provenance performance [15, 16, 20, 21]. Data from these trials can be used to investigate adaptive and genetic variation among populations (e.g. [19, 22, 23]). Based on their genetic, morphological or physiological population characteristics, ecological adaptation models predict best performing provenances and thereby support the delineation of seed zones that are suitable for reforestation and restoration management [14, 24]. Different modelling approaches have been used for such purposes, including purely generic clustering [22, 25], climate envelope models (e.g. [14]), transfer functions (e.g. [26, 27]), genecological models [21] and regression trees [28].

Most studies focus on the performance of test provenances, while less attention has been paid to the pre-selection of provenance regions [29]. In the work of Rehfeldt et al. [30], the importance of a climate-based approach to provenance selection in the face of climatic changes is highlighted. Based on the findings of Wei et al. [22], the authors classified “climatypes” of Douglas-fir populations in North America. When local adaptation occurs, optimal climatic habitat conditions for a population vary from those of other populations farther away [1]. So does a model that represents the varying relation between the climatic environment and a species’ occurrence. The issue of spatial variability in modelled relations is referred to as (spatial) non-stationarity [31, 32]. Most common modelling approaches assume stationarity and represent the relation between model predictors and model response *globally*: the model estimates are constant throughout the complete spatial distribution. The assumption of stationarity, however, is not met when intra-specific variation occurs [31, 33]. In contrast, modelling this relation *locally*, model coefficients vary across space and thus represent the individual-environment linkage for each observation [31, 32]. If a priori genetic information is available, one can separately analyse sub-populations (e.g. [33–38]). Without such information, two methods commonly used to capture non-stationary model relations are spatially varying-coefficient models (SVCMs) and geographically weighted regression (GWR) (both approaches reviewed and compared in [39]).

In this study, we use a non-stationary modelling approach, specifically a SVCM, to identify ecotypes of a wide-spread species based only on climatic distribution data. We do this for Douglas-fir, whose occurrence data are available for its entire North American range. Ecotypes, *sensu* Turesson [40], are varieties of a species which show genotypic similar responses to a specific habitat. Our analysis is based on the assumption that environmental adaptations can be an early stage of genetic differentiation (e.g. [21, 41]). Our aim is to demonstrate how this approach can aid identification of populations to be sampled for climate-tolerance related genetic profiling. The method is based on the assumption that ecotypic variations of Douglas-fir in responses to climate are reflected in the model coefficients. We hypothesize that clustering of these model coefficients yields tentative ecotypes from which provenance regions can be inferred. To test these emerging ecotypes, we compare their distribution to six DNA classes as delineated by Rehfeldt et al. [13].

## Methods

### Study distribution and data

Analysis was carried out with a dataset that contains the complete distribution of Douglas-fir throughout Canada, USA and Mexico in form of binary coded presence and absence observations (Fig. 3a). It consists of 73,932 records of which 18,601 are presences and 55,331 absences. The data set was assembled by the USDA Forest Service and supplied to us by G.E. Rehfeldt ([13], and pers. comm.). Presence-absence data were collated from a network of forest inventory and research ground plots of Mexican, Canadian and US American organizations (CONAFOR, CFS, FIA, respectively). Non-forested areas have been presented by a random sample of data points, used in North American vegetation analysis [13, 42]. According to their geographic location, each presence observation in the data set has been assigned to one of six DNA classes, which correspond to genetics-based large-scale provenance regions (Fig. 3b; [13]). The assignment was done by Rehfeldt et al. [13] and is based on well-documented knowledge and supported by the genotypic classification of a preceding study carried out by Wei et al. [22]. The data provided along with [13] contains information on 44 populations from large-scale genotypic regions in Douglas-fir that were used to investigate the intervarietal phylogeographic history in the original study [22]. In [13] however, they were used to assign the 18,601 presences to six DNA classes and that we in this work use for qualitative comparison with our ecotypic regions.

Climatic and bioclimatic variables were generated for each presence and absence location (reference period: 1961-1990) with the ClimateNA v5.10 software package, available at http://tinyurl.com/ClimateNA, based on the PRISM methodology described by Wang et al. [43].

### Model selection

SVCMs are computationally expensive, which is why we fit the model using only three uncorrelated climate variables that have been identified before as being the main drivers for the climatic niche of Douglas-fir [18]. The spatially-varying coefficient Generalized Additive Model (GAM) was substantially more computer intensive than an ordinary Generalized Linear Model (GLM) (in our case by a factor of 10,000). Pre-selecting a manageable number of (uncorrelated) predictors thus is advisable also in situations where the ecological niche is less well known than in our study species. Climate predictors and their squares, e.g. temperature difference and squared temperature difference, were standardised to mean 0 and standard deviation 1 before being fit. The use of second polynomials in a regression model allows for non-linear effects of predictors on the response, as we would expect for climatic niches [44].

### Spatially varying-coefficient model

We build a spatially varying-coefficient model (SVCM) through a generalized additive model (GAM) [45, 46] as implemented in the *mgcv* package for the R programming environment [47]. The GAM was chosen due to its straightforward implementation of SVCMs [46]. Extending the class of GLMs by allowing specification of (semi-parametric) smooth-functions $f_{j}$ that can be imposed on a set of predictors $x_{1}, \ldots , x_{k}$, a generalized additive model for logistic regression can be written as

1 $$\begin{aligned} g(E(Y)) = \beta _0 + \sum \limits _{j=1}^{p} f_{j}(x_{j}), \end{aligned}$$

with the expected value of the response variable $\mu = E(Y)$, transformed by the link function $g(p) = \ln (\frac{p}{1-p})$ for logistic regression, and an intercept $\beta _0$ [45]. To implement spatially variable coefficients, the smooth functions are specified as functions of equidistant longitude (*x*) and latitude (*y*) of each datum, {$x_{x}, x_{y}$}, in km. These were centered on the origin of the coordinate system for more robust estimation [48]. In the model setup, {$x_{x}, x_{y}$} were multiplied by the covariates of interest [39, 49]. Thus, the SVCM looked like this:

2 $$\begin{aligned} g(E(Y)) = f_{\beta _0}(x_\text {x}, x_\text {y}) + \sum \limits _{j=1}^{k} f_{j}(x_\text {x}, x_\text {y})x_{j} \end{aligned}$$

We chose to represent climatic predictors as quadratic functions, with separate spatial smooths for the linear and the quadratic model terms. We thus have the following predictors with spatially variable parameters in the model: a spatial intercept, yearly mean temperature difference (TD), TD$^2$, total summer (May to August) precipitation (PPT_sm), PPT_sm$^2$, mean temperature of warmest month (MTWM) and MTWM$^2$. Gaussian-process splines with (initial) 100 base dimensions were chosen for each term to smooth estimates [50]. Model parameters were fit by maximum likelihood estimation. Predicting the model terms to the presence locations yielded a 18,601 $\times$ 7 matrix with individual model coefficients for each plot where Douglas-fir occurs.

We used two reference models to be able to gauge the effect of spatially variable coefficients: in direct analogy to the SVCM we fit a (stationary) GLM with quadratic terms; just like the GAM fits linear and quadratic effects (where the splines refer only to how these parameters change through space: see eqn 2), so does the GLM. Secondly, we fit a GLM with additional pairwise interactions: if the spatial variation in the estimated coefficients of the SVCM are due to statistical interactions of the climatic predictors, then this model would capture this effect. All models were compared by their Akaike Information Criterion.

### Coefficient clustering

**Fig. 1:**
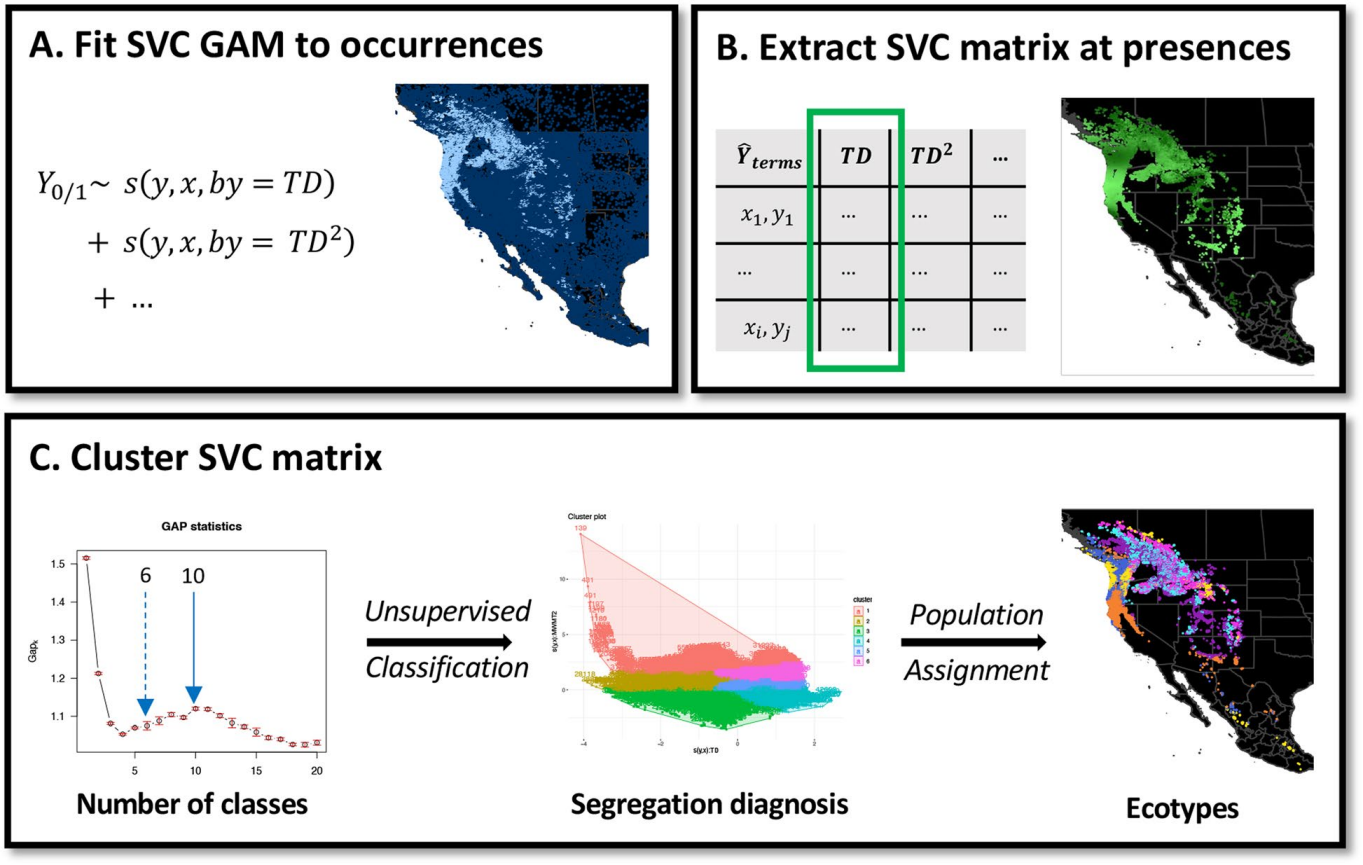
Schematic outline of technical steps from fitting the spatially-varying coefficient (SVC) model to outlining ecotypic regions. A) We fit a GAM to Douglas-fir occurrence data, allowing the smooth terms to vary in space. B) This model predicts occurrences at present populations based on a SVC matrix. C) The coefficients in the SVC matrix are classified by an unsupervised classification algorithm and based on a choice of the optimal amount of clusters, populations are assigned to these ecotypic classes

**Fig. 2:**
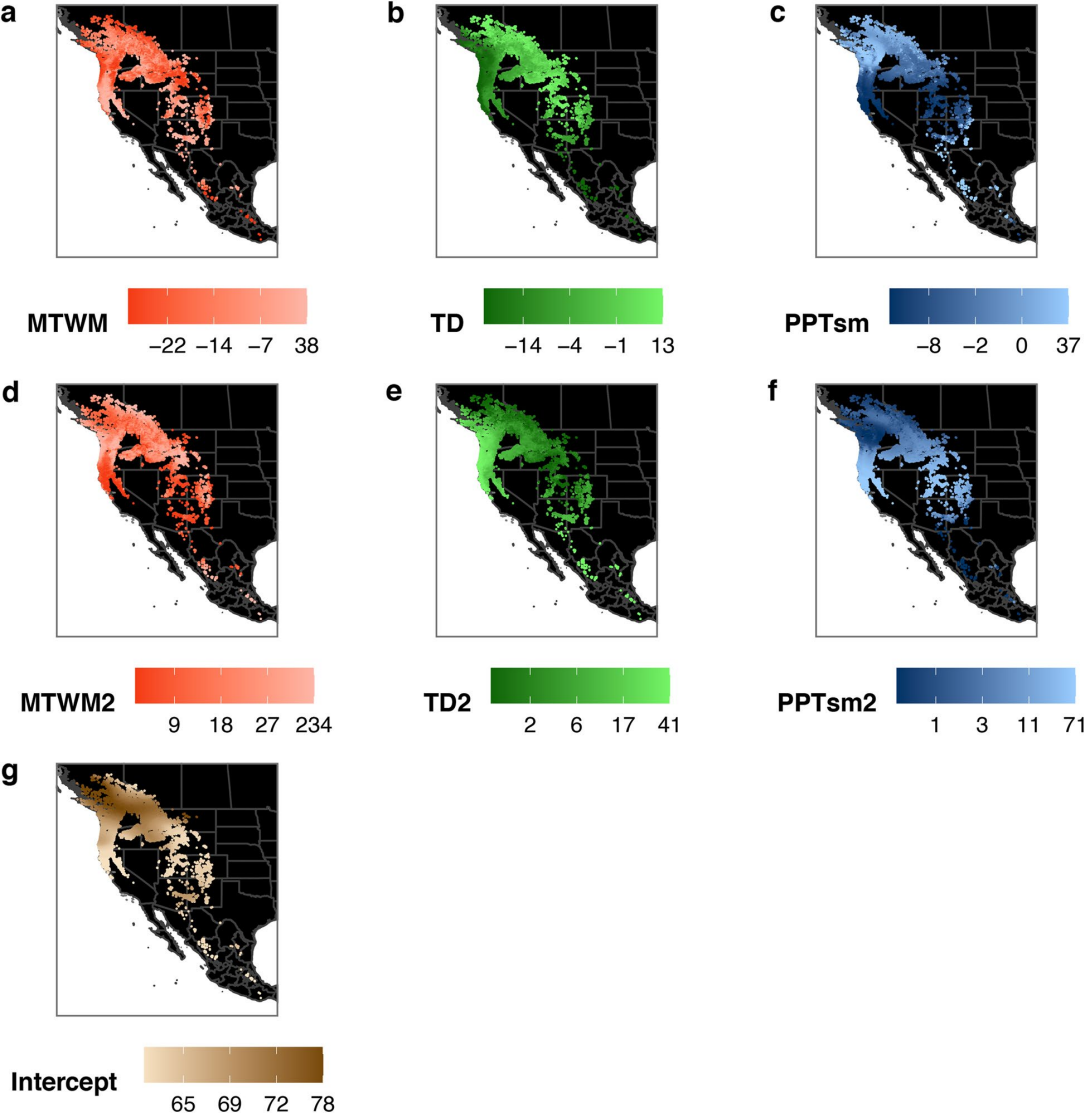
Map of rounded estimates for the effect of the three climatic predictors (top row), their quadratic terms (second row) and the intercept (bottom row). Note that all predictors were standardized before the SVCM was fitted

Ecotypes were defined as groups of populations with similar responses to environmental variables. The statistical model captures this similarities in its spatially-varying coefficients (SVCs) that are therefore implicitly assumed to represent similar ecotypic traits. An unsupervised classification algorithm, namely a fast *k*-medoids clustering algorithm ([51], using R’s cluster::pam) was applied to detect similar subsets in the SVC matrix of presence observations and subsequently assign these observations to ecotypic clusters based on the detected similarity (see Fig. 1 for a schematic overview of where this procedure is embedded in the analysis). In this matrix, estimates for MTWM and the intercept were relatively narrowly distributed, whereas estimates for the effect of TD and PPT_sm exhibited some extreme values. For visualisation, these were 96%-windosorised [52], i.e. extreme values reduced to the second or 98th percentile, resulting in Fig. 2. For clusters of environmental responses however, we omitted the spatial intercept (Fig. 2, panel g), as it only adjusts prevalence estimates. Thus, we used a SVC matrix of 18,601 $\times$ 6 model terms. Correspondence of the detected ecotypes with the six large-scale DNA regions was quantified by the Rand index, $R(C,C') = \frac{2(n_{11}+n_{00})}{n(n-1)}$, where $n_{11}$ is the number of observations both in clusters *C* and $C'$, $n_{00}$ those neither in *C* nor $C'$, and *n* the total number of observations [53].

**Fig. 3:**
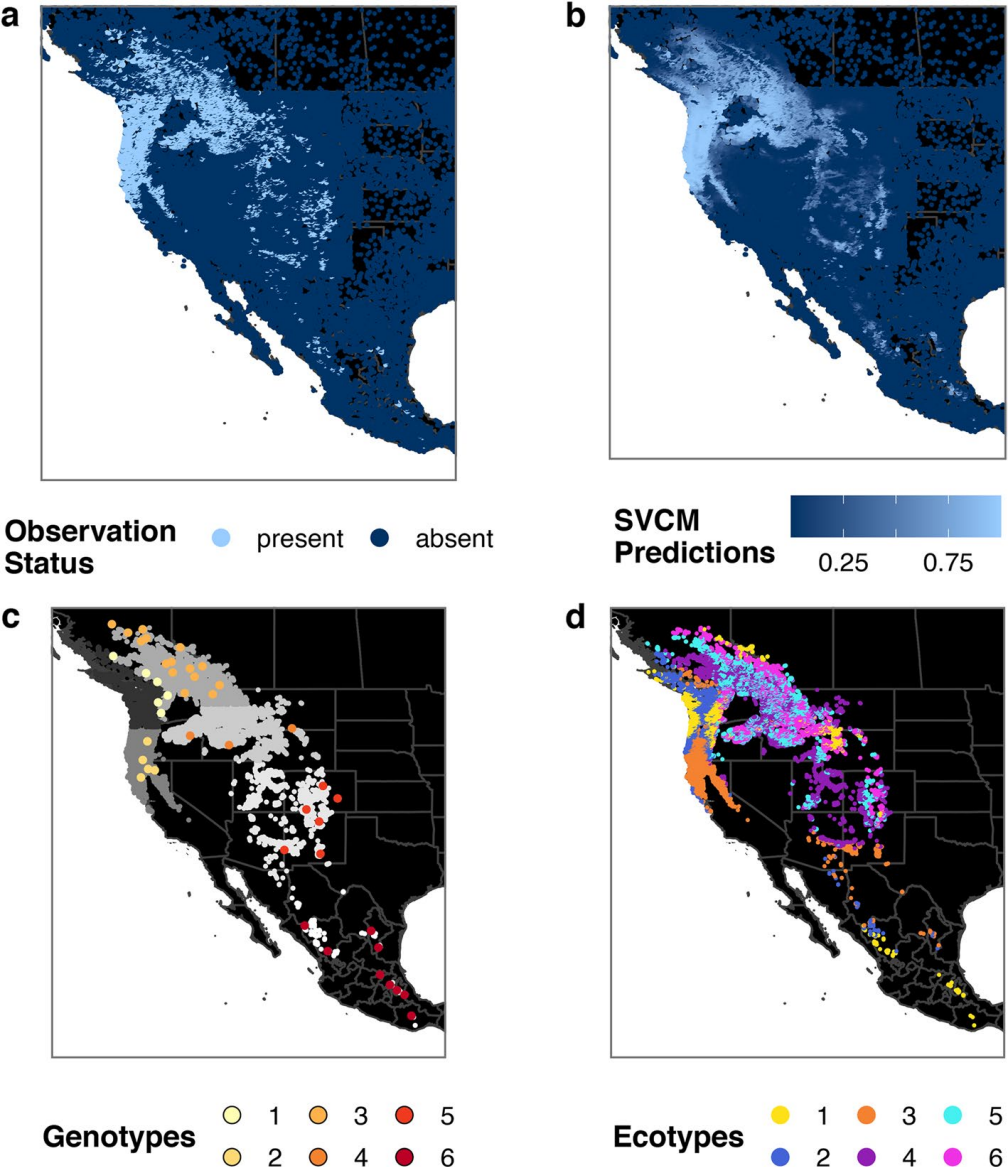
Distribution of Douglas-fir and its deduced ecotypes. Panel **a** displays the presence and absence observations as contained in the data set. Panel **b** shows model predictions for probability of Douglas-fir occurrence, computed by the generalized additive model with spatially variable coefficients. In panel **c**, assignments of observed data points to provenance regions, provided within the data set are mapped in grey tones [13]. The coloured points represent the 44 populations which were genetically analysed and used as baseline for outlining the large scale genotypic regions [22]. Panel **d** shows the assignment of presence data points to one of six ecotypes derived from the model coefficients

Based on the observation that coefficients exhibit very different ranges, we decided to compare *k*-medoids on two different versions of the coefficient matrix: 1. directly on the terms as returned by the model, 2. on the standardised terms (i.e. the correlation matrix), which reduced domination by MTWM estimates. While standardization did not remove the effect of extremes completely, it at least mitigated it (SI, Fig. 1). The resulting clusters differed slightly between these two versions (see Fig. 3 and SI, Fig. 1), but we deem the first approach to be more appropriate for grouping environmental responses, as it keeps the model parameters’ magnitudes, which are directly interpretable as strength of an effect [54].

In the unsupervised classification, we determined the optimal number of clusters using the GAP statistic [55] (SI, Fig. 2). While this indicated as a global optimum without any clusters, the gap statistics identifies a local maximum at ten clusters. We conducted our analysis towards two different ways for validation: (1) the SVC matrix was clustered into six ecotypes to quantify the proposed method with the available large-scale DNA classes.(2) We examined higher resolved ecotypes based on the suggested ten clusters to qualitatively validate these with recent evidence of smaller-scale DNA classes ([14, 25, 56], e.g.). We will discuss the results of the qualitative validation, while corresponding figures can be found in the supplementary information (SI, Qualitative validation of ten ecotypes).

### Cluster differentiation

Variation within and among clusters were assessed by comparing predicted climate niches, depicting individual response norms in marginal conditional effect plots. The effect of a climate predictor on the predicted occurrence was calculated over a sequence from its smallest to its largest value with the other two predictors held constant at the cluster’s mean value. This was done for each observed environmental condition separately, and then averaged across all presence locations of a cluster (also referred to as partial dependence plot). Changes in model prediction for the observation then indicated an effect of the climatic factor on its occurrence.

In order to test for the importance of the Douglas-fir specific clusters, we pursued the cluster analysis also with a “neutral” model, i.e. we fitted the same model with spatially-varying coefficients to randomized presences and absences. Its model terms thus only represent the clustering of climatic predictors across space but carry no Douglas-fir specific information. Similarity of the results was compared with both, the genetic-based provenance regions and the ecotypes.

## Results

The spatially-variable coefficient model (SVCM, Table 1) had a substantially better fit than the stationary GLM with the same structure or a GLM with additional pairwise interactions (Akaike Information Criterion (AIC)$_\text {SVCM}=23439$, AIC$_\text {GLM}=38891$, AIC$_\text {GLM-I}=34694$). It explained 72.4% of the deviance, indicating a very well-fitting model (see also Fig. 3a and b). All three predictors were contributing significantly to the model (see Table 1). Mean temperature of the warmest month (and its square) were the most important model parameters, with $\chi ^2$-values at least four times higher than those of other model parameters (Table 1). The spatial variability of model parameter estimates is depicted in Fig. 2, the averages sizes of these coefficients per ecotype are given in the supplementary information. While in itself hardly interpretable, the result demonstrates clear spatial patterns, with smooth transitions between regions of high and low values. These patterns form the basis for the clustering analysis to identify ecotypes.

The “neutral” spatially-varying coefficent model reported a poor fit with a deviance explained of 0.02% and an AIC of 83431. The model summary can be found in the supplementary information (SI: Table 5).

**Table 1:** Summary of the spatially variable-coefficient model. Edf and Ref.df refer to two different ways to compute the numbers of degrees of freedom absorbed by the spatial spline for a model term. TD, PPT_sm and MTWM refer to temperature difference, summer precipitation and temperature of the warmest month. All $\chi ^2$-values are significant at $p < 0.001$

	edf	Ref.df	$\chi ^2$
s(y,x)	43.76	47.43	330.35
s(y,x):TD	3.00	3.00	127.58
s(y,x):TD2	3.00	3.00	125.35
s(y,x):PPT_sm	32.72	37.25	301.61
s(y,x):PPT_sm2	35.32	39.39	193.43
s(y,x):MWMT	41.68	45.75	1406.34
s(y,x):MWMT2	47.78	51.37	1345.66

### Identification of ecotypes

The six ecotypes identified by *k*-medoids clustering based on the terms of the SVCM exhibit large overlap with the DNA classes (Table 2), but also suggest some deviations. A quantitative comparison of the clustering output to the DNA-classes yielded an agreement of over 75% (Rand index = 0.756, see also Table 2). Ecotypes differed from the genotypes in that, firstly, some are geographically disjunct (ecotypes 1 and 2) and that, secondly, specifically inland they range further than the homogeneous large-scale DNA classes (ecotypes 4 and 5) (Fig. 3, bottom panels).

**Table 2:** Confusion matrix of the 18,601 Douglas-fir presence classifications by DNA (columns) and by coefficient similarity (rows) (percentage of ecotypes assigned to each DNA class in brackets)

	DNA_1	DNA_2	DNA_3	DNA_4	DNA_5	DNA_6
Ecotype 1	1132 (52.8)	509 (23.8)	257 (12.0)	164 (7.7)	8 (0.4)	73 (3.4)
Ecotype 2	1405 (57.1)	986 (40.1)	4 (0.2)	5 (0.2)	9 (0.4)	53 (2.2)
Ecotype 3	135 (4.5)	2580 (86.8)	0 (0.0)	8 (0.3)	211 (7.1)	37 (1.3)
Ecotype 4	70 (1.9)	28 (0.7)	2351 (62.4)	796 (21.1)	520 (13.8)	0 (0)
Ecotype 5	64 (1.6)	29 (0.7)	2305 (57.1)	1500 (37.2)	137 (3.4)	0 (0)
Ecotype 6	61 (1.9)	42 (1.3)	1524 (47.3)	1515 (47.0)	83 (2.6)	0 (0)

With the exception of ecotype 3, clusters do not explicitly accord with only one DNA class (Table 2). In their upper ranges, cluster allocations reveal between 47% accordance (ecotype 6 with DNA class 3) and 86% accordance (ecotype 3 with DNA class 2). While ecotype 6 ranges across the two northern interior genotypic regions (DNA class 3 and 4) nearly equally strong, ecotype 3 covers much of the range of the southern coastal genotype (DNA class 2), with some disjunct but spatially close ecotypic populations in the southern interior region and the northern transition zones.

Another ecotype that is strongly disjunct is ecotype 1 with already two disjunct groups in the northern coastal genotypic region, ranging until the southern coast, and some visible group in the interior north and middel, as well as in Mexico. Ecotype 2 can mainly be found in the northern coastal regions but also ranging than the genotype in this area, namely further south along the coast and further towards the inland regions of DNA class 3. Ecotype 4 shows the largest spread in latitude, from British Columbia over Washington and into the central and southern Rocky Mountains. Overall, these findings show some clear contrasts to the large-scale genetic and taxonomic distinction of coastal and interior subspecies (Fig. 3 bottom left).

Quantifying the similarity of the “neutral” clusters with the DNA classes also yielded an accordance of over 76% (Rand index = 0.763), while they also correspond to over 77% with our ecotypes (Rand index = 0.77) (SI, Fig. 7). This essentially suggests that the SVCM-ecotypes indeed indicate climatic niches.

### Response norms

**Fig. 4:**
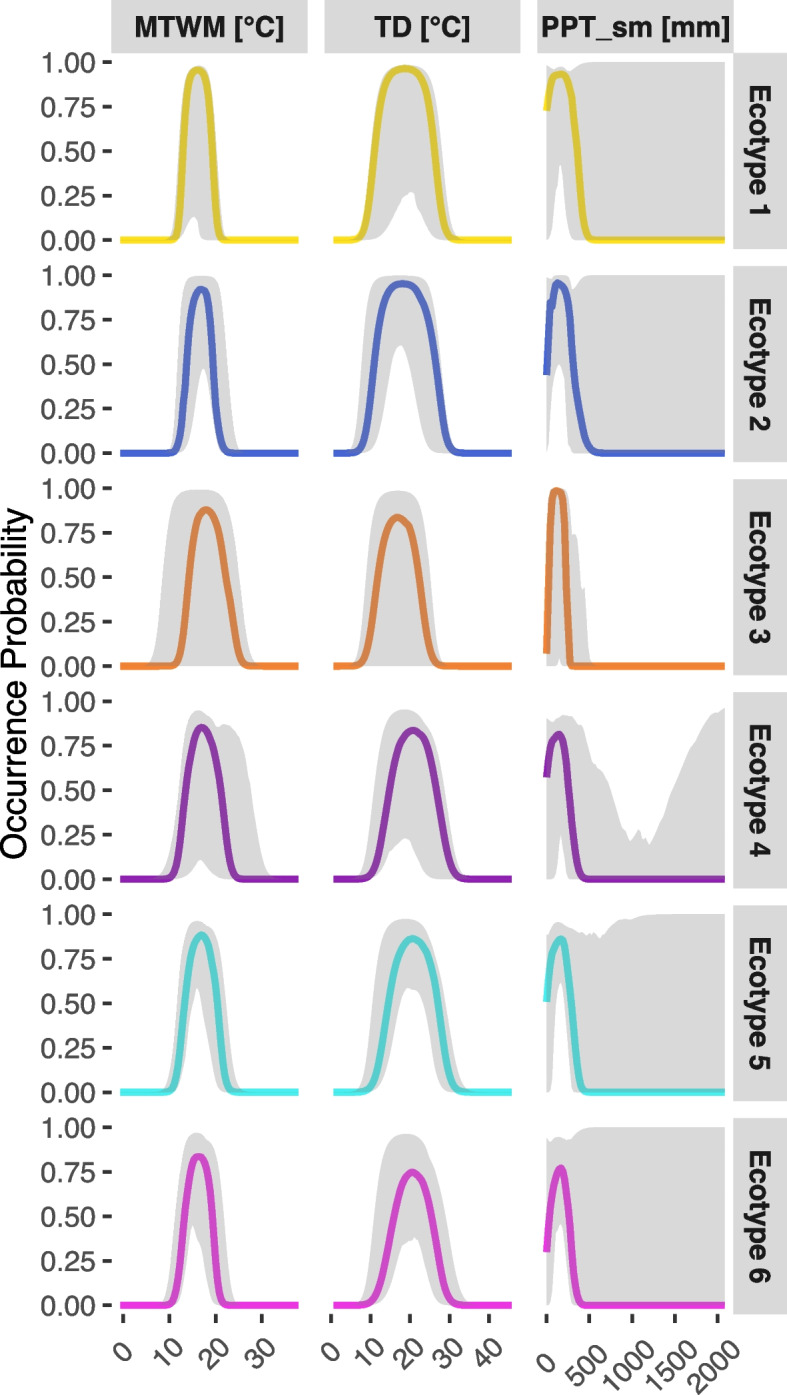
Climate niches of the six clusters identified from model coefficients. Plots are effects of changing the focal predictor while holding the other two at there mean value, for each presence location of that cluster. The median effect (with grey smooth defined by 0.05 and 95% quantiles) is displayed in the ecotypes’ colours. A reduced total size of response indicates a climatic niche with small effect of this predictor on Douglas-fir occurrence

Douglas-fir ecotypes respond to climatic conditions in a similar, yet separable way. The overall climate niche of Douglas-fir seems to be at a temperature optimum around 15°C, with a preference for 10-20°C temperature difference and low precipitation ($< 400$ mm) (Fig. 4). The signal of all three climatic predictors is an occurrence probability larger than 0.75, suggesting that the underlying ecotypes are well constrained by the predictors used. Douglas-fir ecotypes are distinguishable, firstly, by the signal of the climatic predictors, secondly by the variation in that signal and thirdly by its range, i.e. peakedness of the effect curve.

Ecotypes 1 and 2, both mainly found in the northern and southern coastal regions, show a very strong (Table 3) and sharp signal (Fig. 4) over all climatic predictors. The variation in their response to precipitation, however, is large. Not so for the ecotype 3, found mainly in southern coastal regions that specifically for precipitation shows the highest, sharpest and most robust signal. In contrast, the ecotypic responses to MTWM and TD are clearly wider and more variable than their northern coastal friends.

**Table 3:** Maximum conditional median effect size of each predictor on ecotypes, corresponding to the median largest occurrence probability in Fig. 4. The closer the value to 1, the narrower the niche with respect to this variable

	Ecotype 1	Ecotype 2	Ecotype 3	Ecotype 4	Ecotype 5	Ecotype 6
MTWM	0.96	0.92	0.88	0.85	0.88	0.83
PPT_sm	0.93	0.96	0.98	0.81	0.86	0.77
TD	0.96	0.95	0.84	0.84	0.86	0.75

The niche positions of the mainly interior distributed ecotypes are very similar but with a slightly less signal. Further, the variations of their responses have a left (e.g. TD, ecotypes 5 and 6) or right (MTWM, ecotype 4) tendency. Their response to precipitation is poorly constrained by the data. In part, the poorer definition of ecotypes 3 and 6 may be explained by their lower number of records over the larger geographic range (Table 2).

In direct comparison with the map of ecotypes (Fig. 3) it becomes apparent that even disjunct ecotypes of the northern and southern regions (ecotype 4, 5) or eastern and western (ecotypes 1, 2) exhibit rather similar climatic niches with respect to the three predictors under consideration.

## Discussion

Data on the geographic distribution of a species may hold information allowing us to identify groups of populations with similar responses to the environment, as shown here for Douglas-fir and climate. Based on the assumption that trait adaptations to environmental variables are a component of genetic differentiation, we interpret these groups as tentative ecotypes of Douglas-fir [41]. Assigning Douglas-fir populations in their distribution area in North America to six different response groups, ecotypes exhibit different responses characteristics to climate effects, presumably having different climate optima. Quantification of the geographic distribution of the identified ecotypes showed overlap with available large-scale DNA regions to some degree, while qualitative comparison with finer resolved and genetically analysed provenance regions implies ecotypic and genotypic similarities [14, 25, 56]. The results suggest that ecologically induced intra-specific variation in (unobserved) occurrence-related traits, as well as an increased intra-variety differentiation, potentially hybrids, found in transition zones ([57, 58], p. 38) can be detected in a first screening step with a climate-based, local modelling approach to provenance outlining. With this being an explorative approach, the resulting model hypothesises ecotypes which are not validated until compared with genetic information.

### Identified provenance regions

The two main varieties of Douglas-fir, *Pseudotsuga menziesii* var. *menziesii* and *Pseudotsuga menziesii* var. *glauca*, consist of sub-specific populations adapted to certain environments [18]. In allozyme studies it has been shown that beyond variation *among* varieties, there are also intra-varietal allozymic differences as well as clinal variations in both, the coastal and Rocky Mountains sub-species [58, 59]. As such, multiple studies on the genetic differentiation of these two varieties linked genotypes to large-scale regions (e.g. [23, 25, 59, 60]). Allozyme and fossil pollen surveys detected two population sources for the coastal variety, var. *menziesii*, and suggest two to four sources for the Rocky Mountains variety, var. *glauca* [23, 59]. The Mexican populations are mostly treated separately and have even been suggested as an own variety [61]. Although belonging to the subspecies of *Pseudotsuga menziesii* var. *glauca*, particularly the central Mexican populations morphologically and physiologically differ from this variety [61, 62].

Based on the genetic literature, we grouped Douglas-fir populations first into six classes to quantitatively compare deduced ecotypes with available data on large-scale DNA regions [13, 22, 63]. Then we also grouped Douglas-fir populations into ten classes to qualitatively assess their similarities with recent smaller-scale DNA regions [14, 25, 56]. Clustering of the spatially-varying coefficient (SVC) matrix partly reproduced the six DNA class regions without using the DNA-data: we identified spatially well separated, latitudinally arranged, yet disjoint mainly coastal (ecotypes 1, 2 and 3) and interior ecotypes (ecotypes 4, 5, 6). A Mexican ecotype is only indicated but has not been classified as a standalone group. Comparing these ecotypes to the available large-scale DNA classes (Fig. 3c) yields up to a maximum of 86% accordance for the southern coastal variety. Overall, identified ecotypes show much larger ranges, transition into each other and are more fragmented than the strictly outlined DNA class regions, even within the two varieties of *Pseudotsuga menziesii*.

Clustering of the SVC matrix into six groups only approximated the spatial pattern of genetic variation [13]. By allowing for more clusters, which would also be the approach if no DNA-based clusters were previously identified (e.g. [55]), a wider range of ecotypic clusters emerged (see supplementary information), in line with European provenance trials of Douglas-fir and studies on intra-varietal genetic structures [14, 56]. Such a larger number of clusters leads to more spatial compactness in the southern coastal ecotypes and points out an inter-varietal small-scale differentiation in the north (SI, Fig. 5 and SI Tab. 4). An experiment on clustering the SVC matrix separately for the coastal, interior and mexican varieties suggested a fragmentation of the coastal variety into six ecotypes (SI, Fig. 6), which does not fully match a current evidence of four coastal genetic cluster with additional admixed populations [56], but supports its known small-scale differentiation [25]. Douglas-fir is known for its strong local diversity in association to environmental forcing variables [58], of which we only considered three. This might also lead to finer resolved ecotypic regions than considered in our qualitative comparison. In a future work, more predictors could be considered and by dimensional reduction techniques still used in this computationally demanding approach. Dependencies might remain partially incomplete, as selective forces are not restricted to climatic conditions, but also to soil [64], fire [65], pests and so forth [19]. Still, the high spatial coherence of the clusters suggests that the approach as such may have potential that warrants further testing.

### Differentiation of ecotypes

Our ecotypic classification is determined by the interplay of three climatic factors throughout the range of Douglas-fir that might have been responsible for part of the genotypic adaptations in its biogeographic history [66]. These predictors constrain ecotype identification by prior knowledge that is taken from the literature [13]. Douglas-fir, as a highly adaptable species, responds strongly to temperature- and moisture-related changes in its environment ([67], Fig. 4). Gen-ecological studies repeatedly confirmed that intra-specific variation is mainly driven by temperature, specifically winter cold, and by drought, related to summer precipitation [6, 13, 67]. In our analysis, winter temperature was substituted by the highly correlated proxy ‘yearly temperature difference’ (i.e. continentality), while summer temperature and precipitation were directly included in the model. Traits which report adaptation to summer temperature and precipitation change from north to south throughout the distribution of coastal Douglas-fir [60]. Cold hardiness-related traits typically characterize populations of higher elevations and with greater distance to the Pacific ocean, i.e. interior populations [67]. Variation in these traits appears along latitude and elevation [57, 68].

The growth responses of the interior variety, *Pseudotsuga menziesii* var. *glauca*, to climate change scenarios, have been shown to remarkably differ between central and southern interior populations [63]. Our analysis does indeed differentiate within this subspecies, since the three large-scale DNA-regions (Fig. 3c) were also in our analysis subdivided into overall three population clusters, with some fragmented disjunct populations that were assigned to the mainly coastal ecotypes. Increasing elevation of growth sites towards lower latitudes is reflected in rather subtle changes of responses to the temperature related predictors [57]. That might be the reason why climate niches of *Pseudotsuga menziesii* var. *menziesii* ecotypes differ only slightly from those of *Pseudotsuga menziesii* var. *glauca*, i.e. in the strength of the signal and its variation. As known beforehand, precipitation vagary (PPT_sm) and continentality (TD), which characterize the habitat of the coastal and interior varieties respectively [63], reveal large-scale clinal patterns in space (Fig. 2). The effect of summer temperature (MTWM), seems to be the strongest and sharpest for northern coastal ecotypes (1 and 2) and together with a contrasting high variation in precipitation vagary distinguishes them clearly from the southern coastal ecotype 3 (Fig. 4. Southern populations of this variety (corresponding to ecotype 3) are known to suffer from both heavier summer drought and winter precipitation [63] and are also more resistant to drought, while northern populations (corresponding to ecotypes 1 and 2) are more productive [69].

The most glaring difference between botanical knowledge and genetics on one side, and the statistically identified ecotypes on the other, is the disjunct distribution of some subgroups of ecotypes, which cover areas in both coastal and interior range. Also the wide latitudinal range of ecotypes that mainly cover the interior distribution (Fig. 3) is not in line with this knowledge. The subdivision into ecotypes that belong to both, the coastal and interior variety of *Pseudotsuga menziesii* on a small range, such as in British Columbia where we find populations of all ecotypes, indeed happens in a transition zone where we can expect a lot of substructure in populations [57, 66]. This becomes evident when allowing for more clusters in SI, Fig. 4 where the fragmented populations of each variety belong to an own ecotype. Even though local adaptation diversity is known to be very high in Douglas-fir [58], in this area however, is was shown that neural genetic processes shape the differentiation. The wide ranging distributions of our interior ecotypes (4, 5 and 6) do not overlap much with genotypic regions that were classified in other studies [25, 56]. Yet, the disjunct ecotypic subgroup of ecotype 3, of which we find populations in Arizona and New Mexico might point out some of the intra-varietal diversity [25]. However, when applying the cluster algorithm to the coastal and interior varieties separately (see supplementary information), we find strong similarities with the hierarchical genotypic clusters from [25]. While some ecotypes seem to represent higher levels and cover the whole interior distribution, the resolution of ecotypic differentiation in the canadian states as well as in Montana, Wyoming and Idaho is denser and higher (SI, Fig. 7).

The approach used here only identifies ecotypes *with respect to climate*, and is ignorant of other environmental drivers (soil, fire, pests). It is conceivable that the two varieties differentiated by soil preferences overlap substantially in their survival response to climate. Since we analyse occurrence data, and not growth as e.g. Rehfeldt et al. [30], only survival responses matter for the results presented here. Lab research in Douglas-fir seedlings of coastal vs interior provenance found differences in some phenotypic traits, such as growth rates [66], but failed to find difference in others, such as twig water potential response to drought, in CO$_2$ assimilation or stomatal conductance, despite differences in root and leave terpene concentrations [17]. The classification of Douglas-fir populations into six ecotypes by similar responses is somewhat a simplification, as intra-specific variation in adaptation is continuous [13]. Allowing for more ecotypes, for example ten (see supplementary information) did reduce the disjunction of both the coastal and interior variety, but is still limited in representing known interior provenances (SI, Fig. 4).

## Conclusion

Modelling the distribution of a species allowing for spatially variable responses to model predictors allows us to suggest populations that are more similar to another in their response to climate. Since genetic variation is moulded by climate [8], it could prove helpful to pre-select populations for provenance testing by their ecotype-climate niches.

The resulting ecotypes are tentative hypotheses and simultaneously require independent validation, ideally by a combination of genetic and common garden experiments on survival and growth [25]. We propose to use existing occurrence information to inform such sampling as a first screening method (see also [70]).

Ideally, we could follow up on our analysis with sampling of sites with different environmental conditions within ecotypic regions to test for within-cluster homogeneity. In regions with overlap of ecotypes, sampling may need to be more intense (e.g. in Alberta, Canada; Idaho, Wyoming, Arizona, New Mexico, USA; and Durango, Mexico). At the large scale of our analysis, transitions zones and overlaps may also be statistical artefacts of the smooth representation of environmental effects. The strictness of ecotype boundaries cannot be estimated from our data and would require genetic analyses.

Statistical ecotype proposals are likely to improve with the number of data points and finer grain of distribution data. Also, a good prior knowledge of the species’ main environmental constraints helps reducing computational burden. In addition to our example of a commercially interesting tree species, the suitability of this approach can be further investigated at widespread species that are known for sub-specific genetic and morphological differentiation, such as red deer (*Cervus elaphu*s) [71], wolf (*Canis lupus*) [72] or barn owl (*Tyto alba*) [73].

## Supplementary Information

Supplementary Material 1.

## Data Availability

The datasets used and/or analysed during the current study are available from the corresponding author on reasonable request. The code for the analysis can be found here: https://github.com/MWesselkamp/Spatially-varying-coefficient-model.

## Abbreviations

AIC: Akaike Information Criterion
GAM: Generalized Additive Model
GLM: Generalized Linear Model
GWR: Geographically weighted regression
MTWM: mean temperature of warmest month
PPT: sm total summer (May to August) precipitation
SVCM: Spatially varying-coefficient model
TD: yearly mean temperature difference
