# Supplementary material for: Identifying potential provenances for climate-change adaptation using spatially variable coefficient models
Source: BMC Ecol Evol. 2024 May 28;24:70. doi: 10.1186/s12862-024-02260-z (PMC11587631; doi:10.1186/s12862-024-02260-z)
Supplement: Supplementary file 1 — Supplementary Material 1. [file 12862_2024_2260_MOESM1_ESM.pdf]

Identifying potential provenances for climate-change adaptation using spatially variable coefficient models: Supporting Information

List of Figures

1 Correlation plot of the three climatic predictors: mean warmest monthly temperature (MWMT), yearly temperature difference (TD), mean summer precipitation (PPT_sm). **Source: Created by MW** 2

2 GAP statistics with an increasing number of clusters, computed with k-means on the full spatially-varying coefficient matrix of presences. **Source: Created by MW** 3

3 Observation assignments to ecotypes for a preceding choice of 6 clusters based on a standardized coefficient matrix. **Source: Created by MW** 5

4 Observation assignments to ecotypes for a preceding choice of ten clusters, based on the non-standardized coefficient matrix. Quantification of the similarity to the six large-scale DNA classes with Rand's index yielded an agreement of over 75%. **Source: Created by MW** 7

5 Conditional effect sizes for and ecotypic classification with ten clusters. **Source: Created by MW** 8

6 GAP statistics for the two varieties and the mexican populations separately applied. **Source: Created by MW** 9

7 Variety-based ecotypic classification. **Source: Created by MW** 10

8 Coefficient clusters of a "neutral" model, i.e. the same model with spatially-varying coefficients but randomized presences and absences. Its model terms only represent the correlation of predictors across space but carry no Douglas-fir specific information. These clusters can therefore be understood as purely climatic clusters. Quantification of the similarity with Rand's index to ecotypes yielded an agreement of over 76 % for six, and over 76% for ten clusters. **Source: Created by MW** 11

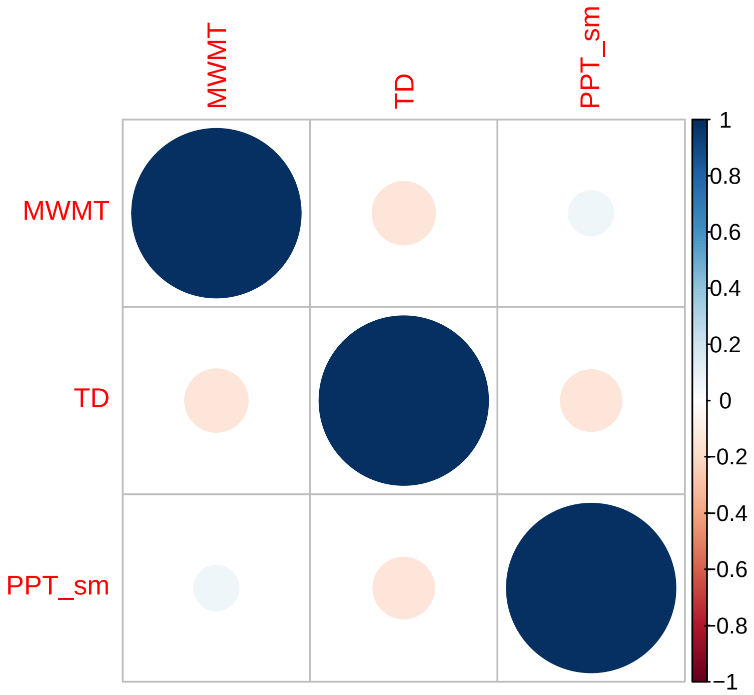

Figure S1: Correlation plot of the three climatic predictors: mean warmest monthly temperature (MWMT), yearly temperature difference (TD), mean summer precipitation (PPT_sm).

Quantitative validation of six ecotypic regions

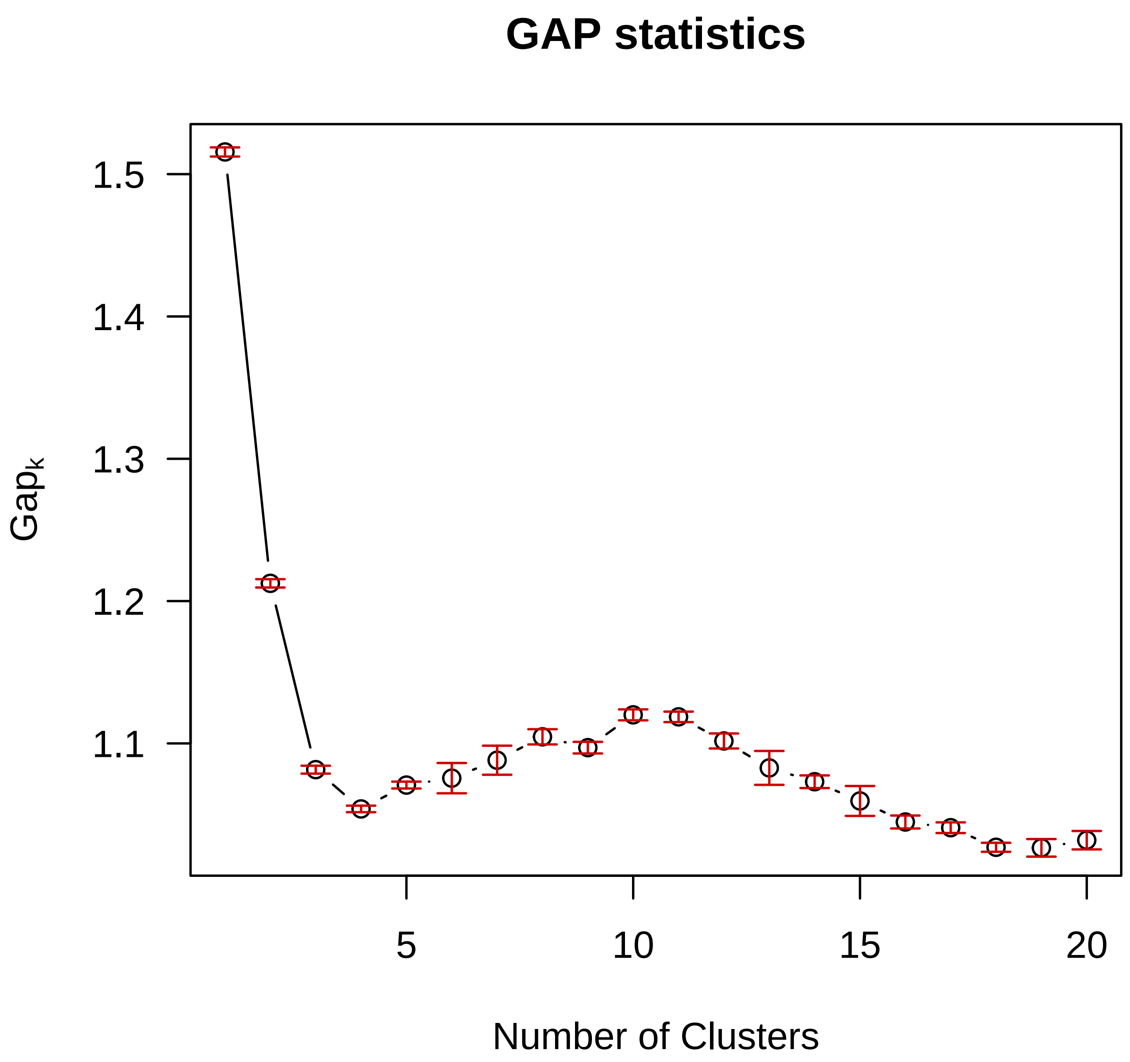

Figure S2: GAP statistics with an increasing number of clusters, computed with k-means on the full spatially-varying coefficient matrix of presences.

Table S1: Confusion matrix of the SVC model with the two main varieties and the mexican populations. Here, we see a clear distribution of Ecotypes 1-3 to the coastal variety and of 4-6 to the inland variety, while the mexican populations are represented only by disjunct groups from coastal ecotypes.

	Coastal	Inland	Mexican
Ecotype 1	1641	429	73
Ecotype 2	2391	18	53
Ecotype 3	2715	219	37
Ecotype 4	98	3667	0
Ecotype 5	93	3942	0
Ecotype 6	103	3122	0

Table S2: Mean and standard deviation of the spatially-varying model coefficients, taken from the SVC matrix and averaged over ecotypic populations.

	s(y,x)	s(y,x):TD	s(y,x):TD2	s(y,x):PPT_sm	s(y,x):PPT_sm2	s(y,x):MWMT	s(y,x):MWMT2
1	65.99±5.13	-14.00±6.8	17.18±7.08	-1.38±5.13	3.35±6.17	-36.99±8.69	47.65±11.13
2	68.17±5.24	-16.49±4.13	19.55±4.37	-3.53±6.94	6.94±10.71	-16.06±5.2	23.02±5.86
3	58.41±10.48	-14.21±4.76	19.52±4.62	-14.91±9.01	29.95±16.9	1.77±7.83	1.68±8.16
4	71.23±4.3	0.13±3.06	2.24±3.74	-2.99±4.95	6.19±8.3	-4.69±4.94	8.19±4.57
5	70.69±3.5	-1.07±2.83	3.58±3.31	-2.42±3.42	4.97±5.51	-14.38±3.33	19.33±2.98
6	68.73±3.64	-1.91±3.37	4.58±3.99	-2.45±3.41	5.06±5.32	-24.17±4.46	30.60±4.61

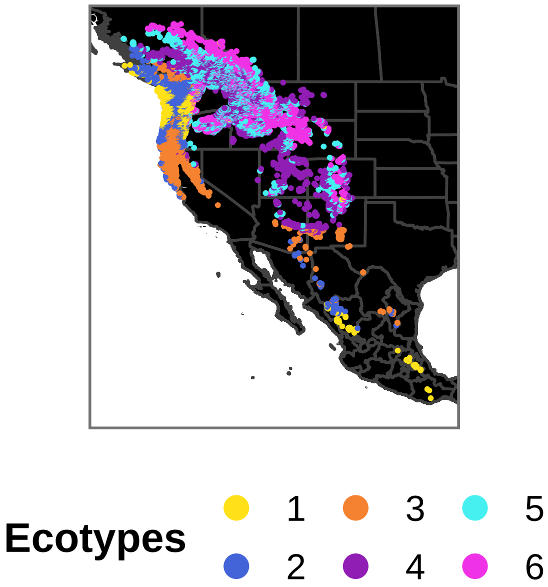

Figure S3: Observation assignments to ecotypes for a preceding choice of 6 clusters based on a standardized coefficient matrix. The Rand index computed from comparision with large-scale DNA classes is 0.759.

Qualitative validation of ten ecotypic regions

Table S3: Confusion matrix of the 18,601 Douglas-fir presence classifications into ten clusters by DNA and by coefficient similarity.

	DNA_1	DNA_2	DNA_3	DNA_4	DNA_5	DNA_6
Ecotype 1	584	209	178	75	2	62
Ecotype 2	812	482	30	2	0	26
Ecotype 3	1072	701	2	1	9	31
Ecotype 4	230	1622	2	0	95	43
Ecotype 5	2	1103	0	14	121	1
Ecotype 6	3	10	903	362	344	0
Ecotype 7	85	19	1725	570	204	0
Ecotype 8	33	14	1602	931	83	0
Ecotype 9	28	10	1202	1273	63	0
Ecotype 10	18	4	797	760	47	0

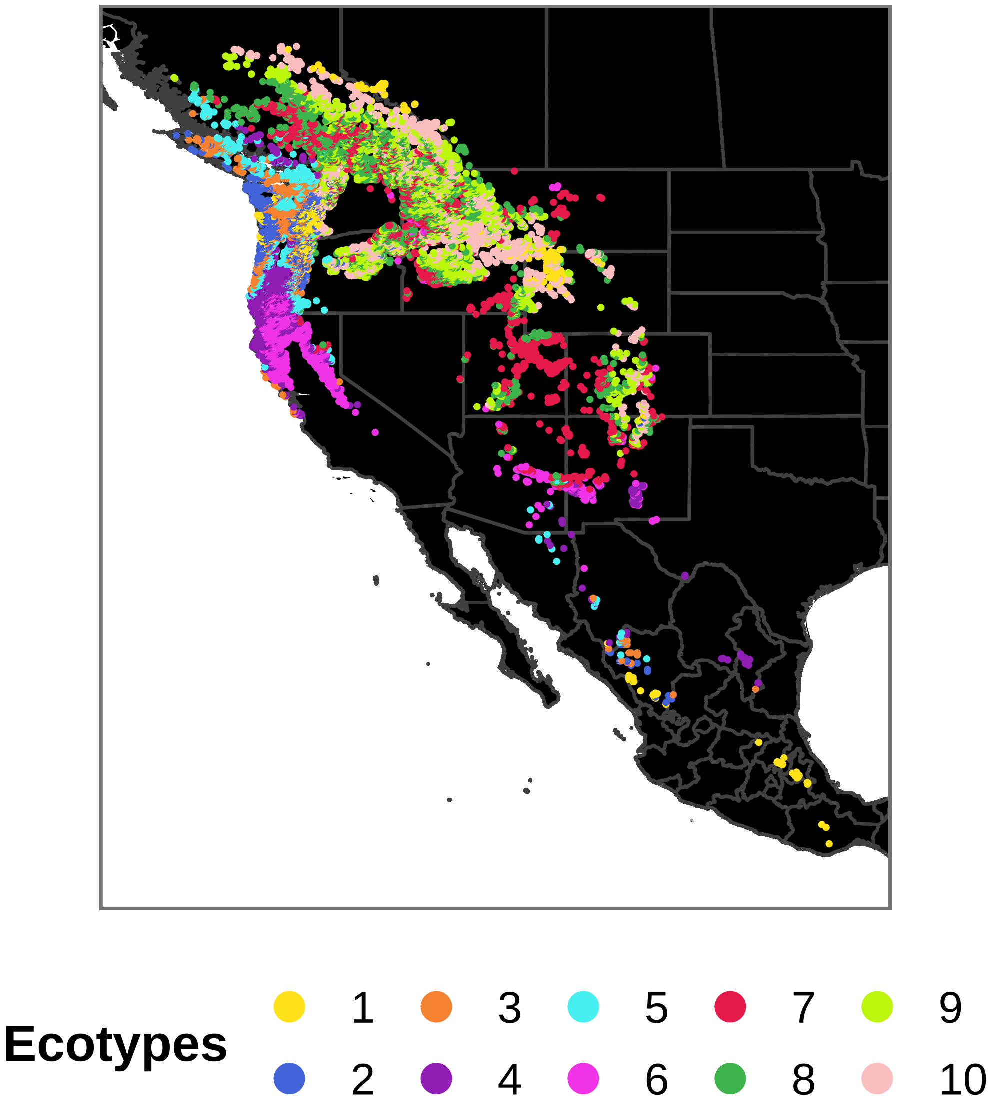

Figure S4: Observation assignments to ecotypes for a preceding choice of ten clusters, based on the non-standardized coefficient matrix. Quantification of the similarity to the six large-scale DNA classes with Rand's index yielded an agreement of over 0.75%.

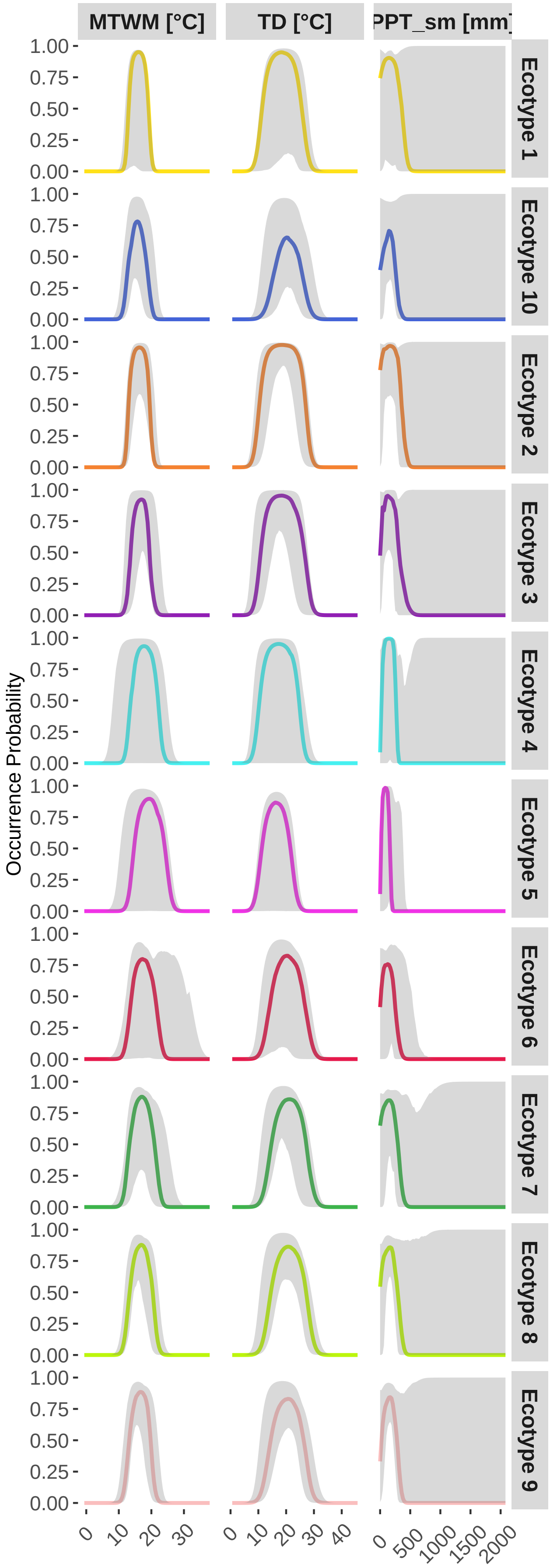

Figure S5: Conditional effect sizes for and ecotypic classification with ten clusters. Colored lines indicate the median response of the ecotype at the mean of the other predictors. Gray smooths indicate the 5% and 95% prediction interval.

Variety-based ecotypic classification

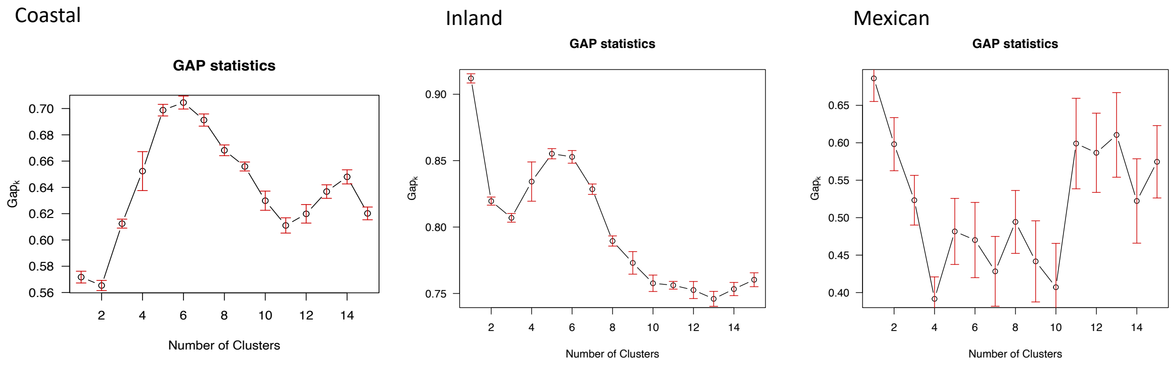

Figure S6: GAP statistics for the two varieties and the mexican populations separately applied. **Left**: Coastal DNA classes (see main text, DNA classes 1 and 2). **Middle**: Interior DNA classes (see main text, DNA classes 3, 4 and 5). **Right**: Mexican DNA class (see main text, DNA class 6). The global maximum indicates an optimal choice of cluster numbers, followed by potential local maxima.

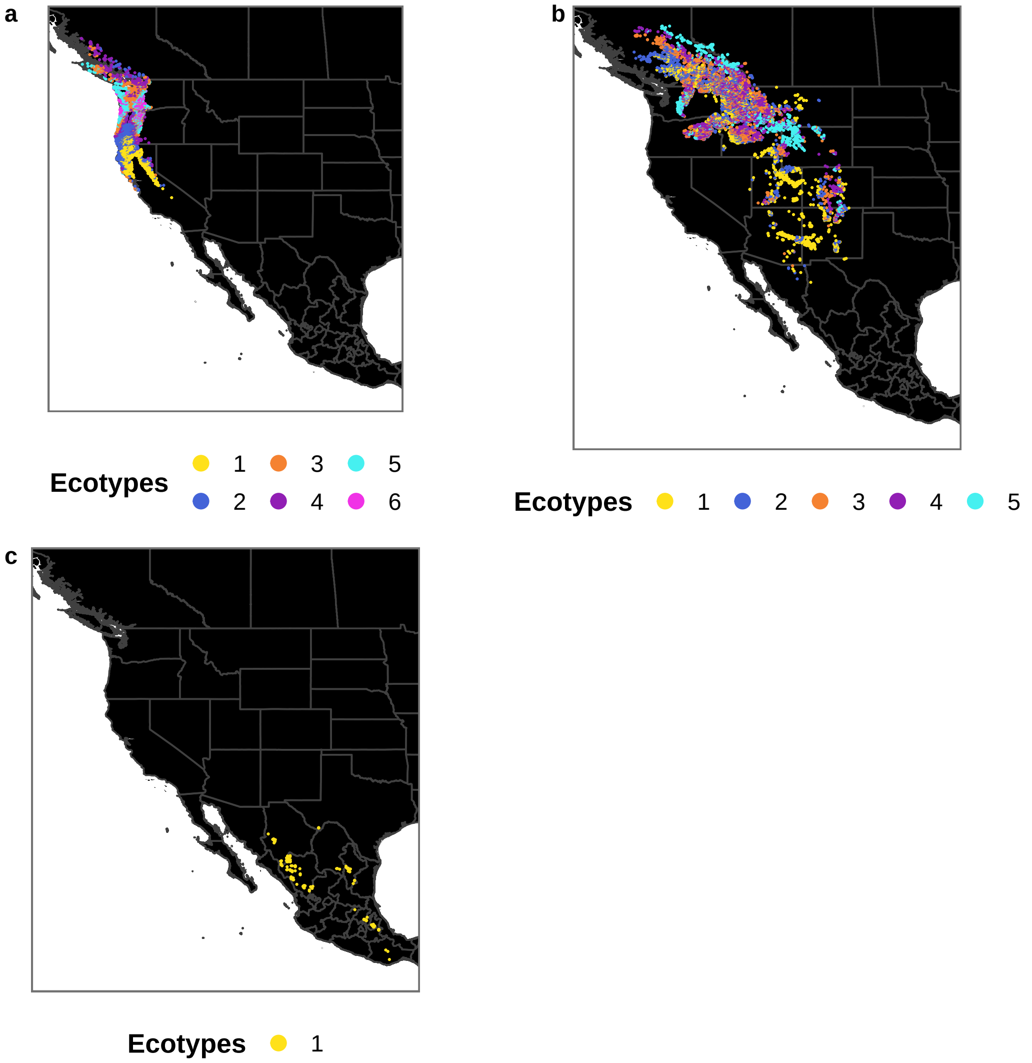

Figure S7: Variety-based ecotypic classification: a) coastal variety, based on the GAP statistic classified into six ecotypes (global optimum), b) interior variety, based on the GAP statistic classified into 5 ecotypes (local optimum) and c) mexican populations, due to the small sample size and GAP statistics grouped as one.

Neutral model

Table S4: Model summary of the neutral spatially-varying coefficient model that was fit to randomized presence and absences of Douglas-fir. It self-reported explained deviance is 0.02%, the AIC is 83431.84 and the R^2 is zero.

	edf	Ref.df	Chi.sq	p-value
s(y,x)	2.00	2.00	0.78	0.68
s(y,x):TD	3.00	3.00	3.40	0.33
s(y,x):TD2	3.00	3.00	3.62	0.31
s(y,x):PPT_sm	3.00	3.00	2.74	0.43
s(y,x):PPT_sm2	3.00	3.00	6.72	0.08
s(y,x):MWMT	3.00	3.00	0.55	0.91
s(y,x):MWMT2	3.00	3.00	0.42	0.94

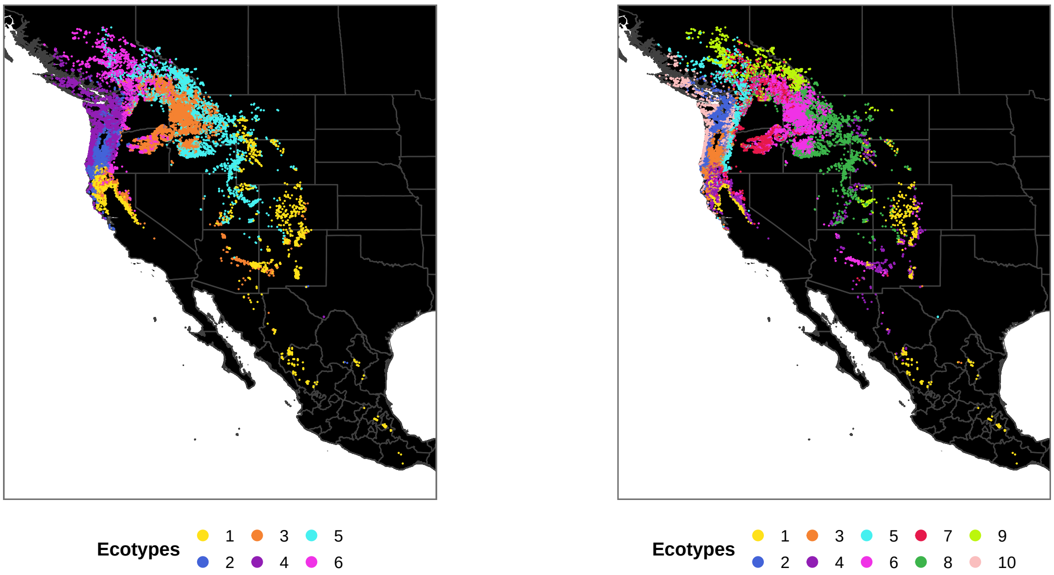

Figure S8: Coefficient clusters of a “neutral” model, i.e. the same model with spatially-varying coefficients but but randomized presences and absences. Its model terms only represent the correlation of predictors across space but carry no Douglas-fir specific information. These clusters can therefore be understood as purely climatic clusters. Quantification of the similarity with Rand’s index to ecotypes yielded an agreement of over 76 % for six, and over 76% for ten clusters.

Table S5: Confusion matrix of the neutral model.

	1	2	3	4	5	6	7	8	9	10
1	0	1124	157	2	219	1	40	0	0	1324
2	260	528	1437	652	615	71	201	0	0	410
3	0	0	0	1	823	1730	1296	747	1827	17
4	39	0	8	97	31	1482	1085	1237	9	0
5	257	0	2	324	0	126	5	217	37	0
6	133	1	2	21	6	0	0	0	0	0
